# Peptide-Based Physical Gels Endowed with Thixotropic Behaviour

**DOI:** 10.3390/gels3040039

**Published:** 2017-10-21

**Authors:** Nicola Zanna, Claudia Tomasini

**Affiliations:** Dipartimento di Chimica “Giacomo Ciamician”—Alma Mater Studiorum Università di Bologna—Via Selmi, 2-40126 Bologna, Italy; nicola.zanna2@unibo.it

**Keywords:** drug delivery, injectable hydrogels, peptides, peptidomimetics, regenerative medicine, rheology

## Abstract

Thixotropy is one of the oldest documented rheological phenomenon in colloid science and may be defined as an increase of viscosity in a state of rest and a decrease of viscosity when submitted to a constant shearing stress. This behavior has been exploited in recent years to prepare injectable hydrogels for application in drug delivery systems. Thixotropic hydrogels may be profitably used in the field of regenerative medicine, which promotes tissue healing after injuries and diseases, as the molten hydrogel may be injected by syringe and then self-adapts in the space inside the injection site and recovers the solid form. We will focus our attention on the preparation, properties, and some applications of biocompatible thixotropic hydrogels.

## 1. Introduction

Thixotropy is one of the oldest documented rheological phenomena in colloid science [1]. Although it is very common in industrial and natural systems, a general rheological model capable of fully describing the different features of thixotropy has not yet been developed, as there is even some confusion about its definition.

This phenomenon was described for the first time in 1923 by Schalek and Szegvari [2,3], when it was observed that a solution is formed when a hydrogel based on Fe_2_O_3_ is shaken, but a sol-gel transition occurs if the mixture is allowed to rest. After that, a lot of other materials displaying a similar behavior were discovered, so rheology became a distinct discipline and a definition of thixotropy was coined: “The application of a finite shear to a system after a long rest may result in a decrease of the viscosity or the consistency. If the original viscosity or consistency is recovered, this behavior is called thixotropy” by IUPAC.

The word thixotropy comes from Ancient Greek θίξις thixis “touch” and -τρόπος -tropos “of turning” and was originally invented by Herbert Freundlich for a sol-gel transformation [4,5]. Detailed reports of the early history can be found in the reviews by Bauer and Collins [6], Mewis [7], and Barnes [8].

At present, thixotropy is defined as the continuous decrease of viscosity with time when flow is applied to a sample that has been previously at rest and the subsequent recovery of viscosity in time when the flow is discontinued. The essential elements of the definition used nowadays are that:

(i) it is based on viscosity;
(ii) it implies a time-dependent decrease of the viscosity induced by flow;
(iii) the effect is reversible when the flow is decreased or arrested.

Recently, stimuli-responsive materials showing dramatic property changes in response to external environmental stimuli [9] have been attracting considerable attention due to their wide ranges of potential applications in biomaterials, sensors, displays, surface science, etc. [10,11]. Among them, stimuli-responsive supramolecular organogels and hydrogels are one kind of the most attractive examples [12,13,14,15,16,17,18,19,20,21].

**Figure 1:**
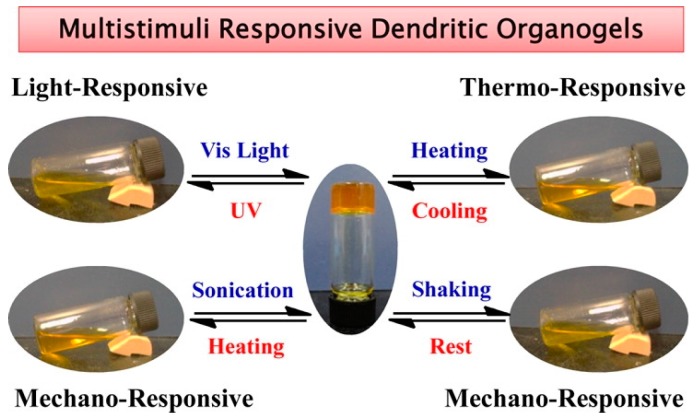
Gel–sol and sol–gel transitions of an organogel in 2-methoxyethanol. Image adapted with permission from reference [10]. Copyright 2012 American Chemical Society.

Low molecular weight gelators (LMWGs) [22,23] assemble into physically cross-linked three-dimensional networks, so the solvent molecules are entrapped through noncovalent weak interactions, as H bonding and π−π stacking. External triggers, as temperature, light, chemicals, and/or mechanical force, can easily modulate their physical behaviors (Figure 1), due to the weak nature of these forces [24,25,26,27,28,29].

Hydrogels are materials of scientific interest for innovative applications in materials chemistry, biology, and medicine. The high-water content of hydrogels makes them useful for mimicking the extracellular matrix (ECM) by creating a three-dimensional support in which cells can grow, or as a carrier for drug delivery with controlled release [30,31]. Among the properties needed for these applications, biocompatibility is necessary, while great attention is devoted to the rheological and thixotropic behavior. Thixotropic hydrogels include polymeric hydrogels and molecular hydrogels. Polymeric hydrogels are formed by crosslinking polymers [32,33], whereas molecular hydrogels are formed by the self-assembly of small molecules with a molecular weight of less than 2000 DA via non-covalent interactions.

In this article, we want to present an overview of the most recent results for the preparation of hydrogels endowed with thixotropic behavior. Then we will describe some examples of applications of thixotropic hydrogels as materials for the culture and encapsulation of cells. They may be injected to act locally in the specific region which is being treated, avoiding surgical procedures.

## 2. Thixotropic Hydrogels Prepared Using Biocompatible Low Molecular Weight Gelators (LMWGs)

Supramolecular hydrogels, formed through the noncovalent assembly of low molecular weight gelators (LMWGs), are materials of scientific interest for innovative applications in biomedicine [34,35], catalysis [36], and materials chemistry [37]. Because of their reversible character, water gelation by small molecules provides gel-to-sol transitions and a rapid response to external stimuli [38,39]. Appropriate design and the limited synthetic effort required in their preparation allow one to prepare molecules able to control the formation of assembled structures and their properties on the macroscopic level in high yields. This control is highly desirable for practical applications because it enables the creation of hydrogels with tunable mechanical properties [40,41].

We will show here some examples of thixotropic hydrogels with interesting properties that have been obtained using protected amino acids or small peptides, all containing an aromatic moiety (to form π−π stacking interactions) and a polar moiety able to form hydrogen or electrostatic bonds. All those contributions allow the formation of fibrils and fibers that trap the solvent, resulting in a gel formation. We have selected some examples of small molecules that efficiently behave as LMWG, forming strong and thixotropic hydrogels.

Lysine (K) is a peculiar amino acid, bearing a primary amine at the end of an aliphatic chain. When it is derivatized with aromatic moieties, it easily forms supramolecular fibers and hydrogels, through the formation of hydrogen bonds and π–π stacking interactions.

**Figure 2:**
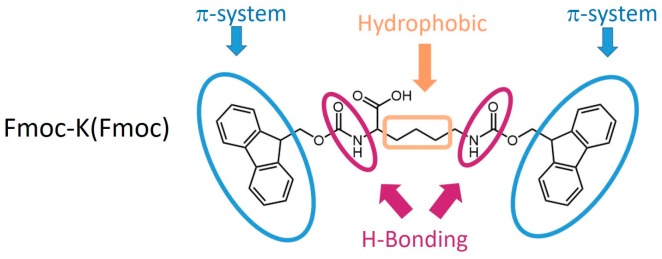
Chemical structure of the gelator Fmoc-K(Fmoc). The highlighted regions indicate the corresponding possible interactions during self-assembly.

In the first paper [42], the authors demonstrated that double Fmoc functionalized l-lysine amino acid [Fmoc-K(Fmoc)] formed gels (Figure 2), whereas a single Fmoc-functionalized l-lysine (Fmoc-K) failed under similar experimental conditions. The second Fmoc-moiety provides a lead to construct or design new synthetic Fmoc-based LMW organogelators and/or hydrogelators [43,44].

**Figure 3:**
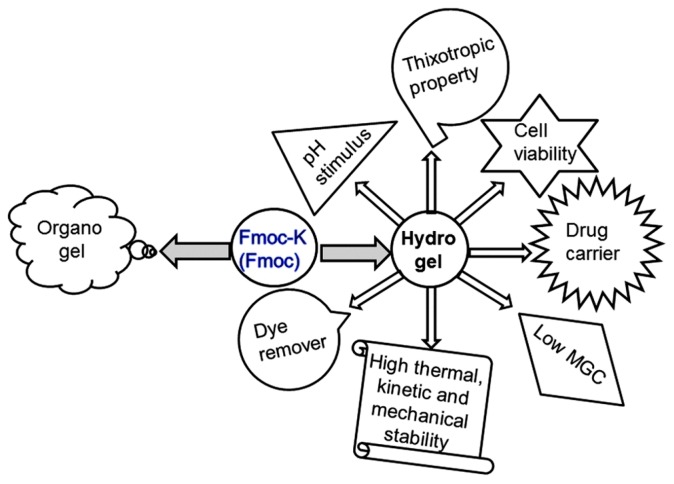
This cartoon diagram summarizes all the possible applications of the hydrogels prepared with the Fmoc-K(Fmoc) gelator. Image adapted with permission from reference [42]. Copyright 2015 Royal Society of Chemistry.

Fmoc-K(Fmoc) gelator has several advantages as it induces both pH-controlled hydrogelation and organogelation (pH-controlled ambidextrous gelation). This property is limited among the gelators, as they predominantly form either hydrogels or organogels. Additionally, the Fmoc-K(Fmoc) hydrogels may be prepared with a very low gelator concentration (MGC = 0.1 wt %). These hydrogels exhibit high thermal stability (about 100 °C), excellent thixotropic properties, and high mechanical strength. The cartoon reported in Figure 3 summarizes the applications of Fmoc-K(Fmoc), presenting a smart gelator that is employed in different applications in applied sciences, including drug delivery, tissue engineering, hazardous dye removal, etc.

**Figure 4:**
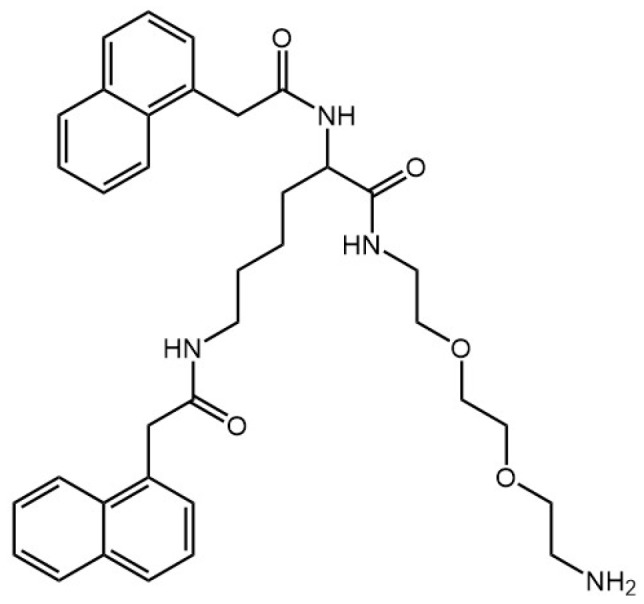
Chemical structure of the amphiphilic gelator derived from lysine.

Another amphiphilic gelator derived from lysine has been reported [45]. Its structure consists of a naphthalene moiety at the N termini and an ethyleneoxy unit with free primary amine at the C terminus (Figure 4).

**Figure 5:**
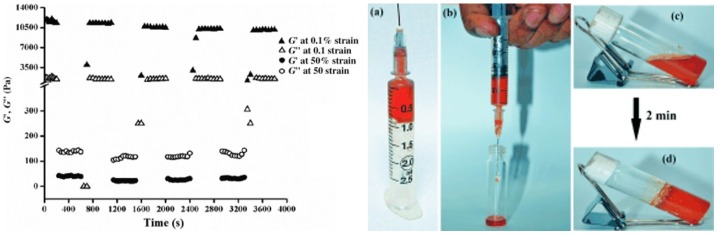
(**Left**) Time-dependent repetitive cycle of step-strain analysis of the hydrogel–AgNP soft nanocomposite with a 0.6 % *w/v* gelator concentration. (**Right**) Photographs of (**a**) AgNP-incorporated hydrogel in a syringe; (**b**) nanocomposite gel flowing through the needle of a syringe; (**c**) solution of AgNP-1 composite after syringe processing; and (**d**) AgNP-including hydrogel that is re-formed after shear thinning at room temperature for 2 min. Image adapted with permission from reference [45]. Copyright 2014 John Wiley and Sons.

This gelator allows gel formation with a minimum gelation concentration of 0.6% *w/v* in mixtures of dimethyl sulfoxide and phosphate buffer at pH 7.4. The hydrogel was characterized by spectroscopic and microscopic studies to study the role of non-covalent interactions in self-assembly gelation. Then Ag nanoparticles were synthesized in the hydrogel by the in situ photo-reduction of AgNO_3_, in which the gelators act as reducing/stabilizing agents. Rheology of the soft nanocomposite showed significant mechanical strength and thixotropic self-recovery properties, which made the composite suitable for use as a syringe-injectable hydrogel that exhibited excellent antibacterial activity against both Gram-positive and Gram-negative bacteria, low hemolytic activity, and high biocompatibility to mammalian (Chinese hamster ovarian) cells. Moreover an agar–gelatin film infused with these nanocomposites allowed the normal growth of mammalian cells on its surface. The thixotropic property of the gel nanocomposite prepared at a physiological pH made it suitable to be utilized as an injectable hydrogel (Figure 5), as previously reported by Schneider et al. [46].

**Figure 6:**
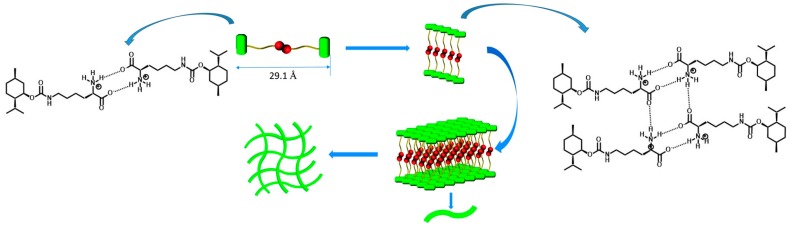
The simulative diagram of the self-assembly process of the hydrogelator based on (−)-menthol. Image adapted with permission from reference [47]. Copyright 2014 Royal Society of Chemistry.

A novel hydrogelator based on (−)-menthol and l-lysine has been designed and synthesized (Figure 6) [47]. It forms a stable hydrogel with a thixotropic character in a large pH range. Moreover, the viscoelastic character of the hydrogel can be enhanced by mechanical force. As a result, the hydrogelator can gelate aqueous solutions of some confirmed antibacterial agents such as Zn^2+^ and a series of water soluble organic antibiotic medicines like lincomycin, amoxicillin, etc. The hydrogel can be developed as a universal carrier for antibacterial agents and may also be widely used in the fields of cell culture, tissue engineering, or drug delivery systems [48,49].

**Figure 7:**
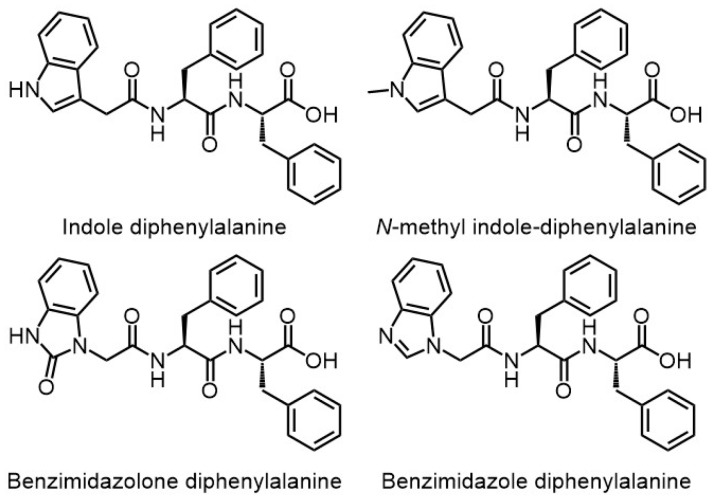
Chemical structure of FF-derived hydrogelators.

Martin et al. synthesized four diphenylalanine-based peptides (Figure 7) that differ for the heterocyclic *N*-protecting groups [50]. Gelation was monitored for these four peptides through zeta potential and electrical impedance spectroscopy measurements: the impedance data show different gelation times for each peptide hydrogel. The relationship between the mechanism of hydrogels self-assembly and their macroscopic behavior was established through atomic force microscopy and rheological measurements: the nitrogen substitution degree affects the self-assembly mechanisms of the hydrogels and there is an interplay between branching and bundling self-assembly pathways that are responsible for the final properties of each hydrogel.

**Figure 8:**
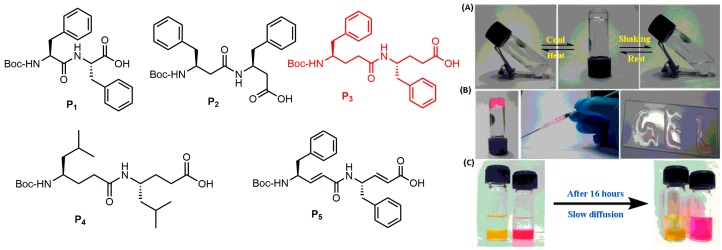
(**Left**) Chemical structures of peptides **P1**−**P5**. (**Right**) (**A**) Stimuli-responsive behavior of peptide **P3** hydrogels; (**B**) Injectable nature of peptide **P3** hydrogels; (**C**) Slow release of proflavine (yellow) and rhodamine (pink) from hydrogel matrix to the buffer layer after 16 h. Image adapted with permission from reference [51]. Copyright 2017 American Chemical Society.

Very recently, the spontaneous supramolecular assembly of a backbone engineered γ-peptide scaffold and its utility in the immobilization of semiconductor quantum dots and in cell culture has been reported [51]. This γ-peptide scaffold efficiently gelates both aqueous phosphate buffers and aromatic organic solvents. A comparative and systematic investigation reveals that the greater spontaneous self-aggregation property of γ-peptide over the α- and β-peptide analogues is mainly due to the backbone flexibility, increased hydrophobicity, and π−π stacking of γ-phenylalanine residues. The peptide hydrogel has displayed a stimuli-responsive and thixotropic signature, which leads to the injectable hydrogels (Figure 8). 2D cell culture studies using normal and cancer cell lines reveal the biocompatibility of γ-peptide hydrogels.

**Figure 9:**
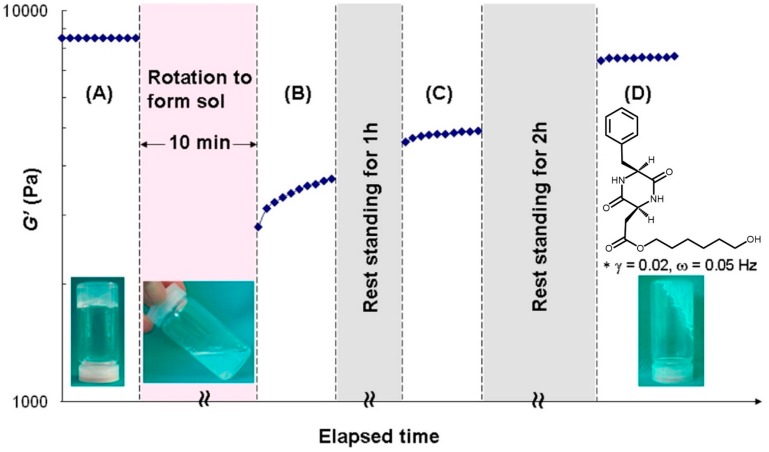
Time-dependence of the storage modulus. The sample was prepared from *cyclo*(l-*O*-hydroxyhexylaspartyl-l-phenylalanyl) in an ethanol/water mixture (20% ethanol/80% water) at 20 g·L^−1^. Image adapted with permission from reference [52]. Copyright 2013 American Chemical Society.

Hoshizawa, Hanabusa, and coworkers reported new hydrogelators and their behavior during gel−sol−gel transitions [52]. Cyclo(l-*O*-hydroxyhexylaspartyl-l-phenylalanyl) is a gelator that forms a thermally/isothermally reversible physical gel with several protic solvents, as water, saline, alcohols, 1.0 M aqueous NaCl, KCl, CaCl_2_, and MgCl_2_ solutions (Figure 9). TEM observations show self-assembled fibers with diameters of 10−100 nm that may be disrupted by a shear stress. The thixotropic behavior is due to the disruption of the van der Waals forces between the alkylene chains under shearing, confirmed by the FT-IR results, which revealed that the gels were formed by hydrogen bonding and van der Waals forces. These results were repeatedly and reproducibly observed at room temperature, even when measurements were repeated many times.

**Figure 10:**
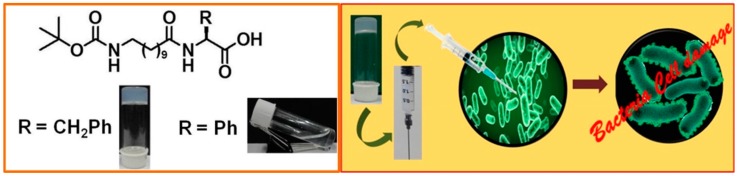
Chemical structures of the peptides and an illustration of the injectable nature of the hydrogel. Image adapted with permission from reference [53]. Copyright 2016 American Chemical Society.

Finally, the dipeptides reported in Figure 10 form a hydrogel in phosphate buffer with a pH level ranging between 6.0 and 8.8 [53]. The hydrogel formed at pH 7.46 has been characterized by small-angle X-ray scattering (SAXS), wide-angle powder X-ray diffraction (PXRD), Fourier transform infrared (FT-IR) spectroscopy, field-emission scanning electron microscopy (FE-SEM), high-resolution transmission electron microscopy (HR-TEM) imaging, and rheological analyses. Moreover, the hydrogel may be injected as it exhibits thixotropic behavior at pH 7.46 and good antibacterial activity against Gram-negative bacteria *Escherichia coli* and *Pseudomonas aeruginosa*. The authors observed that a small change in the molecular structure of the gelator peptide not only turns the gelator into a non-gelator molecule under similar conditions, but also has a significant negative impact on its bactericidal character.

**Figure 11:**
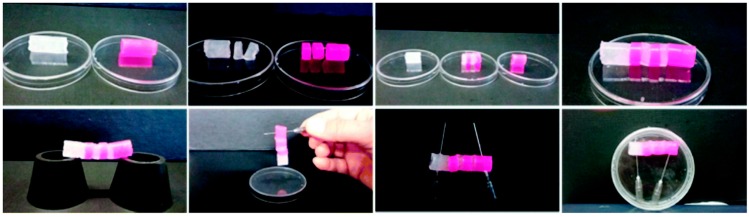
(**Top**) The four images show the sequence followed to prepare the hydrogel bridge (length ≈ 4.0 cm). (**Bottom**) The four images demonstrate that the hydrogel has a self-healing property. The right-end image was taken after one week. Image adapted with permission from reference [57]. Copyright 2016 Royal Society of Chemistry.

Our group recently reported the formation of thermoreversible and/or thixotrophic hydrogels induced by the self-assembly of bolamphiphilic or Fmoc-protected pseudopeptides [54]. Several agents have been tested as self-assembly triggers and pH regulators: pH variation due to the slow hydrolysis of glucono-δ-lactone (GdL) [55,56], or the addition of an amino acid [57] or metal cation [58]. The resulting gels have been characterized by the measurement of the melting points (T_gel_), transparency, gelation time, and viscoelastic properties, together with ECD analysis. Physical characterization of hydrogels was carried out by a morphologic evaluation and rheological measurements and demonstrates that the analyzed hydrogels possess self-heling (Figure 11) and thixotropic properties.

**Figure 12:**
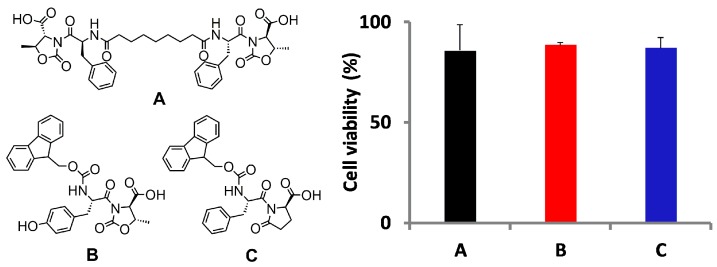
(**Left**) chemical structure of the gelators A–C, studied in this work. (**Right**) viability of embedded HGFs in hydrogels A, B, and C after seven days of culture. Data were expressed as relative percentage ± SD compared to control HGFs. Image adapted with permission from reference [59]. Copyright 2016 American Chemical Society.

Pseudopeptides containing the D-Oxd or the D-pGlu [Oxd = (4*R*,5*S*)-4-methyl-5-carboxyl-oxazolidin-2-one, *p*Glu = pyroglutamic acid] moiety and selected amino acids were used as low molecular weight gelators (LMWGs) to prepare strong and thixotropic hydrogels at a physiological pH (Figure 12). The addition of calcium chloride to the gelator solutions induces the formation of insoluble salts that are organized in fibers at a pH close to the physiological one [59]. As these hydrogels are easily injectable and may be used for regenerative medicine, they were biologically assessed by cell seeding and viability tests. Human gingival fibroblasts (HGFs) were embedded in 2% hydrogels: all the hydrogels allow the growth of encapsulated cells with a very good viability. The toxicity of the gelators may be correlated with their tendency to self-assemble, and is totally absent when the hydrogel is formed.

**Figure 13:**
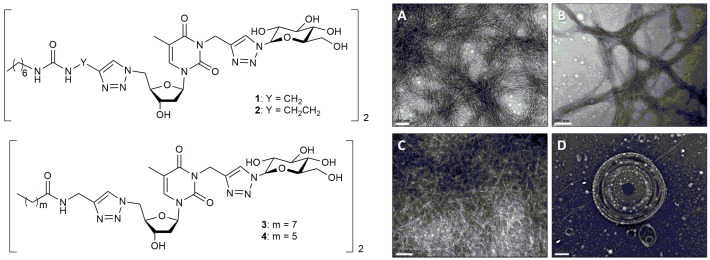
(**Left**) Chemical structures of bolaamphiphiles **1**, **2**, **3,** and **4**. (**Right**) TEM images of aqueous samples at 2% *w/v* obtained for bolaamphiphiles (**A**) **1** (scale bar: 100 nm), (**B**) **2** (scale bar: 100 nm), (**C**) **3** (scale bar: 100 nm)**,** and (**D**) **4** (scale bar: 200 nm). Image adapted with permission from reference [60]. Copyright 2017 Elsevier.

An example of the application of bolamphiphilic peptides to the formation of thixotropic hydrogels has been recently reported [60]. The self-assembly of bolaamphiphiles creates a unique hydrogel supramolecular structure featuring fast gelation kinetics, high elastic moduli, and thixotropic and thermal reversibility properties (Figure 13). Barthelemy et al. evaluated some low molecular weight gelators in vivo and identified one urea-containing molecule that avoids foreign body reactions in mice. This soft material, which inhibits recognition by macrophages and fibrous deposition, exhibits long-term stability after an in vivo injection.

## 3. Applications of Thixotropic Peptide Based Physical Hydrogels

**Figure 14:**
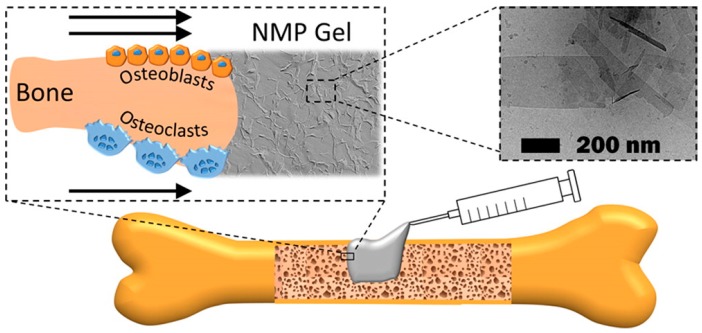
Schematic representation of a nanocrystalline magnesium phosphate (NMP) hydrogel and its application for bone repair. Image adapted with permission from reference [76]. Copyright 2016 American Chemical Society.

Supramolecular hydrogels are used in a range of biological applications, including drug delivery, tissue engineering, and cell culture. Regenerative medicine is a field of increasing interest as it promotes tissue healing after injuries and diseases [61]. Tissue engineering involves the use of biomaterial scaffolds to create in vitro three-dimensional tissue-like structures that simulate the extracellular matrix (ECM) where cells can grow [62,63,64], as often typical bi-dimensional cell cultures lack the ability to effectively simulate the physiological environment. As hydrogels are mainly constituted of water (>95%), they have been extensively studied as materials for the culture and encapsulation of cells [65,66,67,68,69,70,71,72] and may be injected to act locally in the specific region which is being treated [73,74,75], avoiding surgical procedures. The thixotropic behaviour extends the range of hydrogel applications, as it can be easily transferred through a syringe and locally injected. For example, Laurenti et al. recently reported the formation of a thixotropic hydrogel based on magnesium phosphate nanosheets able to accelerate bone healing and osseointegration by enhancing collagen formation, osteoblasts differentiation, and osteoclasts proliferation [76]. This thixotropic, biocompatible, and stable biomaterial is injectable through high gauge needles (Figure 14) and can minimize the invasiveness of orthopedic and craniofacial interventions.

**Figure 15:**
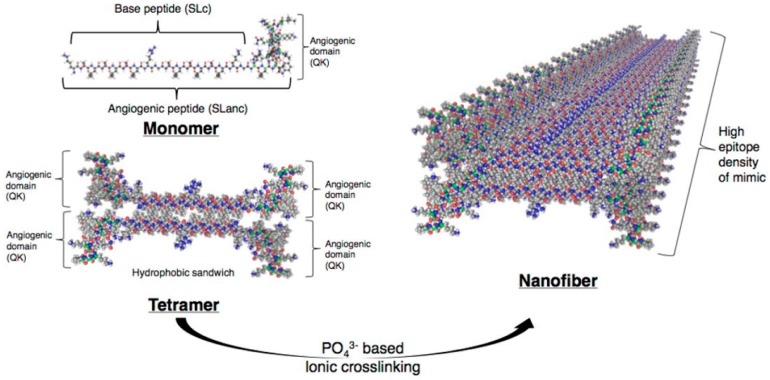
Schematics show how the VEGF mimic polypeptides self-assemble through hydrophobic packing and hydrogen bonding along the fiber axis, exposing the angiogenic domain. Image adapted with permission from reference [77]. Copyright 2016 Elsevier.

Peptide-based hydrogels were successfully used for the revascularization of ischemic tissues [77]. Strategies to promote blood vessel development have capitalized on stem cells and growth factors to favour de novo niches for angiogenesis (Figure 15), with strategies including the delivery of growth factors such as the VEGF protein (vascular endothelial growth factor). Kumar et al. designed a proangiogenic self-assembling VEGF mimic containing angiogenic domain able to form hydrogels. The injection in the ischemic induced the hind limb of mice to promote a mature angiogenic response, favored by the permanency of the growth factor in the region of the injection, due to the solid-like characteristic of the hydrogels endowed with thixotropic properties.

**Figure 16:**
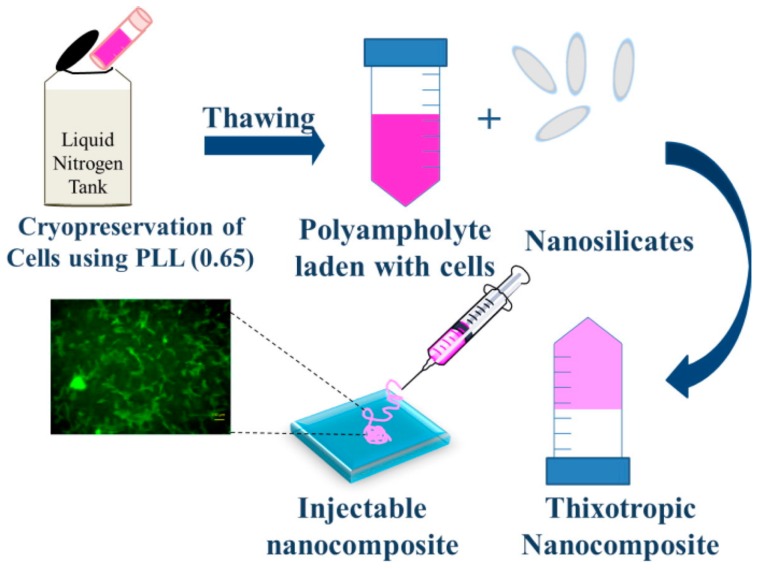
Schematic representation of thixotropic nanocomposite formation from poly-l-lysine and nanosilicates. Image adapted with permission from reference [40]. Copyright 2016 Elsevier.

Another interesting field of application of peptide-based hydrogels is related to cell culture and tissue engineering. As the hydrogels are, by definition, solid-like materials mainly composed by water, the similarity with the extracellular matrix (ECM) becomes obvious. The peptide nature of hydrogels ensures the biocompatibility, hence the possibility to encapsulate various types of cells inside the gel matrix, emulating the three-dimensional environment where cells are used to grow. Jain et al. make use of poly-l-lysine and nanosilicates (Figure 16) to form thixotropic hydrogels able to act as cryopreservation agents, for the purpose of cell delivery after cryopreservation by simple injection into defect sites [40]. This approach does not require any separate cell seeding before injection, thus eliminating the need for cell harvesting and cell maintenance. In this way, cells can be cryopreserved until use; when required, the cells encapsulated in the hydrogel can be revived to their original state and are ready for use.

**Figure 17:**
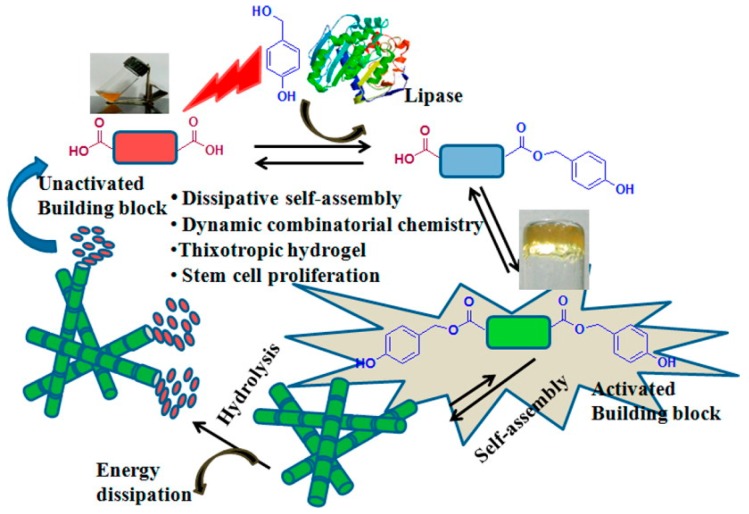
Schematic of gel formation for a bolamphiphile peptide-based hydrogel with self-assembly process mediated by a lipase-catalyzed reaction. Image adapted with permission from reference [78]. Copyright 2015 American Chemical Society.

Das et al. reported the formation of a bolamphiphile peptide-based hydrogel obtained with a self-assembly process mediated by the lipase-catalyzed inclusion of *p*-hydroxybenzylalcohol to peptide bolaamphiphiles [78]. This process generates dynamic combinatorial libraries (DCL) in aqueous medium that mimic the natural dissipative system (Figure 17). The activated diester building block self-assembles to produce nanofibrillar thixotropic hydrogel. The hydrolysis of the ester groups causes the formation of nonassembling bolaamphiphile, resulting in the collapse of nanofibers. This thixotropic hydrogel is used for 3D cell culture experiments for different periods of time: it significantly supports the survival and proliferation of human umbilical cord mesenchymal stem cells.

Jacob et al. also reported a study on several hydrogels based on Fmoc *N*-protected di- and tri- peptides able to encapsulate mesenchymal stem cells and drive their neuronal differentiation, through fibril mediated contact guidance [79].

**Figure 18:**
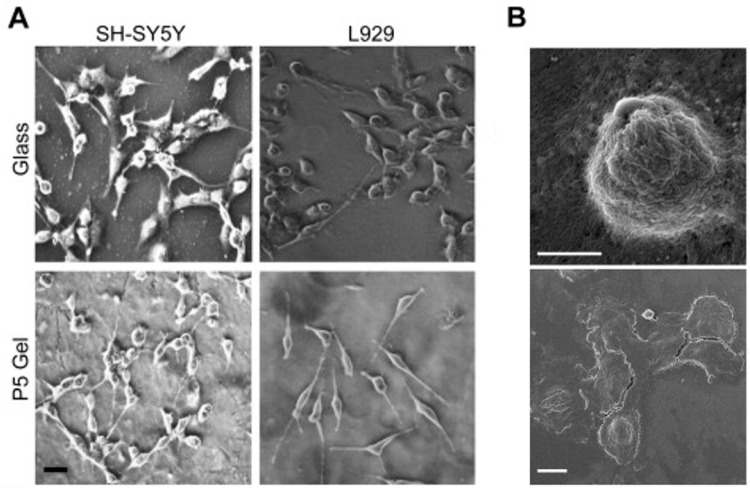
2D cell culture using peptide-based hydrogels. (**A**) Phase contrast images of attached cells of SH-SY5Y cells and L929 cells grown for 24 h on glass substrates and P5 gel. Scale bar for all images are 100 mm. (**B**) SEM image depicting cell adhesion and spreading of SH-SY5Y on P5 hydrogel after 1 h (top) and 24 h (bottom) of incubation respectively. Scale bars are 1 mm (**top**) and 10 mm (**bottom**). Image adapted with permission from reference [79]. Copyright 2015 Elsevier.

Amyloids are highly ordered protein/peptide aggregates responsible for neurodegenerative diseases and various native biological functions. Given the diverse range of physiochemical properties of amyloids, the authors designed a series of peptides based on the high aggregation C-terminus of Ab42, which is associated with Alzheimer’s disease, to prepare novel hydrogels for biomaterial applications. As foreseen, these Fmoc protected peptides self-assemble to β-sheet rich nanofibrils, forming hydrogels with several properties: they are thermoreversible, non-toxic, and thixotropic. These hydrogels help support cell attachment and spreading across a diverse range of cell types (Figure 18).

Finally, silk fibroin- (SF) based materials have been widely studied and applied in bio-related areas due to their excellent structural and biological properties and biocompatibility, hence why their use in the preparation of hydrogels for biological applications, alone or in combination with other materials, is exhibiting an increasing growth. Several papers have recently reported on the application of thixotropic hydrogels based on silk nanofibers. We show here some examples.

**Figure 19:**
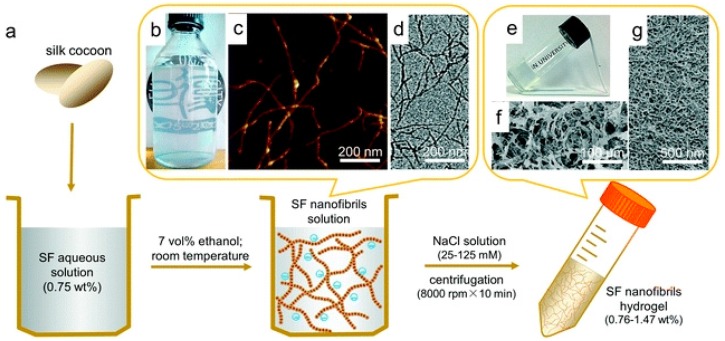
Fabrication of SF nanofibril-based hydrogels with an ECM-like structure. (**a**) Schematic representation of the procedure followed to prepare SF nanofibril-based hydrogels; (**b**) The resultant SF nanofibril solution with opalescence; (**c**,**d**) AFM and TEM images of SF nanofibrils; (**e**) SF nanofibril-based hydrogel; (**f**,**g**) SEM images of lyophilized SF nanofibril-based hydrogel with different magnifications, which show an ECM-like structure. Image adapted with permission from reference [80]. Copyright 2016 Elsevier.

Liu et al. reported the preparation of an injectable hydrogel, obtained by via the simple fibrillation and centrifugation of SF nanofibrils (Figure 19) [80]. These hydrogels resemble an extracellular matrix-like structure, in addition to having good mechanical properties and an outstanding thixotropic character, as the storage modulus (G’) can recover to 93% within 40 s after a large shearing strain (5000%). Finally, the injectable hydrogel exhibits significant biocompatibility for L929 cells cultured in hydrogel after injection.

**Figure 20:**
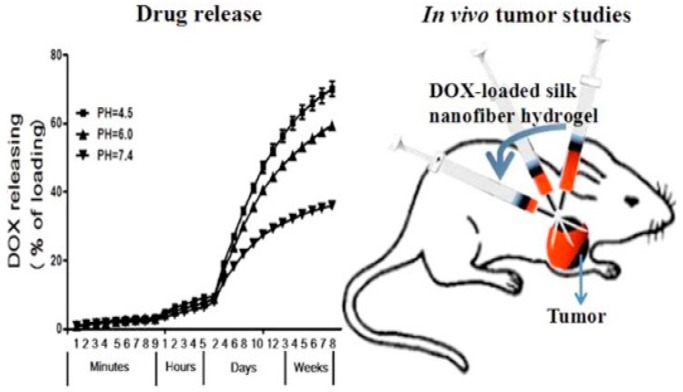
Kinetic of doxorubicin (DOX) release as a function of time. Image adapted with permission from reference [81]. Copyright 2016 American Chemical Society.

Wu et al. reported the preparation of a silk nanofiber-based hydrogel and its use to deliver doxorubicin for localized chemotherapy in breast cancer (Figure 20) [81]. This thixotropic gel, which becomes liquid during injection and then quickly recovers into the solid form after injection, allows the in vivo injection of doxorubicin-loaded gels. This study demonstrates that the doxorubicin-loaded hydrogel outperforms the equivalent dose of free doxorubicin administered intravenously, thanks to the ability of this hydrogel to maintain the shape in the site of injection and gradually release the drug.

**Figure 21:**
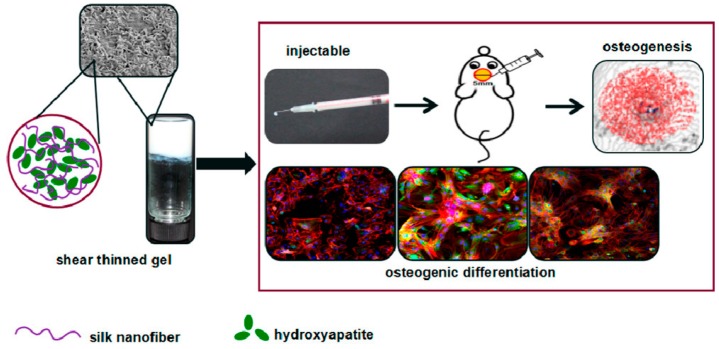
Images of silk- hydroxyapatite hydrogel, its fiber-like structure (**left**) and its application in bone regeneration (**right**). Image adapted with permission from reference [82]. Copyright 2017 American Chemical Society.

Silk-based thixotropic hydrogels incorporating hydroxyapatite (HA) (Figure 21) show osteogenic capability and the ability to favor the regeneration of new bone at damaged sites after an in vivo injection in animal models [82]. This application may become an alternative to autologous bone grafts in the case of bone defects from trauma, abnormalities, non-union fractures, infections, or tumor resection.

**Figure 22:**
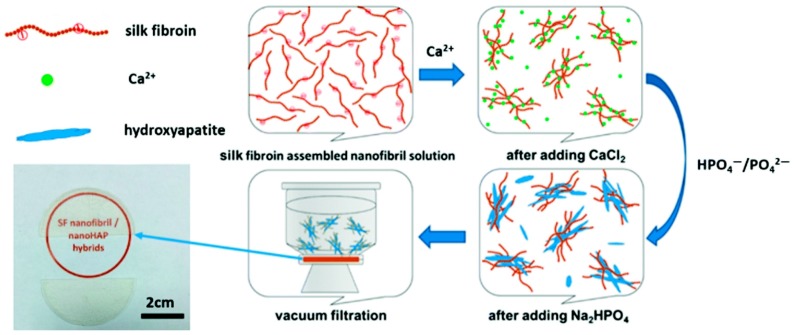
Schematic representation of the procedure followed to prepare silk fibroin nanofibril/nanoHAP films and the resultant photograph. Image adapted with permission from reference [83]. Copyright 2016 Royal Society of Chemistry.

To harvest silk fibroin- (SF) based organic/inorganic composites with various general properties (e.g., hard or soft) and to produce a film or a hydrogel of SF-nanofibril/nanohydroxyapatite, Shao et al. employed the strategies of vacuum filtration and centrifugation (Figure 22) [83]. SF-nanofibril mediated the mineralization of hydroxyapatites (HAP) in situ and the morphology of such organic/inorganic nanohybrids presented a “flower-like” structure, mainly because of the strong interaction between SF-nanofibrils and nanohydroxyapatites. On the other hand, the extracellular matrix (ECM) like SF/HAP hydrogel illustrated not only an adequate mechanical strength, but also a remarkable thixotropy, with the storage modulus (G’) being able to recover to 85% within 50 s when a large shearing strain (5000%) was applied.

## 4. Conclusions

Thixotropy is one of the oldest documented rheological phenomena in colloid science. Although the phenomenon is very common in industrial and natural systems, a general rheological model capable of fully describing the different features of thixotropy has not yet been developed and there even remains some confusion about its definition.

Stimuli-responsive materials showing thixotropic properties in response to external environmental stimuli have attracted considerable attention due to their wide ranges of potential applications in biomaterials, sensors, displays, surface science, etc.

The use of low molecular weight gelators (LMWGs) may lead to the formation of thixotropic supramolecular hydrogels, which are physically cross-linked three-dimensional networks with solvent molecules entrapped inside through noncovalent intermolecular interactions such as H bonding and π−π stacking.

Supramolecular thixotropic hydrogels may be used in a range of biological applications, including drug delivery, tissue engineering, and cell culture. Regenerative medicine is a field of increasing interest as it promotes tissue healing after injuries and diseases. Tissue engineering involves the use of biomaterial scaffolds to create in vitro three-dimensional tissue-like structures that simulate the extracellular matrix (ECM) where cells can grow, as typical bi-dimensional cell cultures often lack the ability to effectively simulate the physiological environment. As hydrogels are mainly constituted of water (>95%), they have been extensively studied as materials for the culture and encapsulation of cells and may be injected to act locally in the specific region being treated, avoiding surgical procedures. The thixotropic behaviour extends the range of hydrogel applications, as it can be easily transferred through a syringe and locally injected.

## References

[1] Mewis J., Wagner N.J. (2009). Thixotropy. Adv. Colloid Interface Sci..

[2] Schalek F.E., Szegvary A. (1923). Ueber Eisenoxydgallerten—Vorlaufige Mitteilung. Kolloid-Z..

[3] Schalek E., Szegvari A. (1923). Die langsame Koagulation konzentrierter Eisenoxydsole zu reversiblen Gallerten. Kolloid-Z..

[4] Goodeve C.F. (1939). A general theory of thixotropy and viscosity. Trans. Faraday Soc..

[5] Reiner M., Scott Blair G.W. (1967). Chapter 9—Rheological Terminology. Rheology, Theory and Applications.

[6] Bauer W.H., Collins E.A. (1967). Chapter 8—Thixotropy and Dilatancy. Rheology, Theory and Applications.

[7] Mewis J. (1979). Thixotropy—A general review. J. Non-Newton. Fluid Mech..

[8] Barnes H.H.A., Barnes A. (1997). Thixotropy—A review. J. Non-Newton. Fluid Mech..

[9] Higuchi A., Ling Q.D., Kumar S.S., Chang Y., Kao T.C., Munusamy M.A., Alarfaj A.A., Hsu S.T., Umezawa A. (2014). External stimulus-responsive biomaterials designed for the culture and differentiation of ES, IPS, and adult stem cells. Prog. Polym. Sci..

[10] Liu Z.-X., Feng Y., Yan Z.-C., He Y.-M., Liu C.-Y., Fan Q.-H. (2012). Multistimuli Responsive Dendritic Organogels Based on Azobenzene-Containing Poly(aryl ether) Dendron. Chem. Mater..

[11] Jones C.D., Steed J.W. (2016). Gels with sense: Supramolecular materials that respond to heat, light and sound. Chem. Soc. Rev..

[12] Weiss R.G. (2009). Preface to the Molecular and Polymer Gels; Materials with Self-Assembled Fibrillar Networks Special Issue. Langmuir.

[13] Terech P., Weiss R.G. (1997). Low Molecular Mass Gelators of Organic Liquids and the Properties of Their Gels. Chem. Rev..

[14] Abdallah D.J., Weiss R.G. (2000). Organogels and Low Molecular Mass Organic Gelators. Adv. Mater..

[15] Sangeetha N.M., Maitra U., Kasagi N., Yamane H., Ojida A., Hamachi I., Maskos K., Reinhoudt D. (2005). Supramolecular gels: Functions and uses. Chem. Soc. Rev..

[16] Dastidar P. (2008). Supramolecular gelling agents: Can they be designed?. Chem. Soc. Rev..

[17] Ajayaghosh A., Praveen V.K., Vijayakumar C., Sommerdijk N.A.J.M., Ajayaghosh A., Meskers S.C.J., Schenning A.P.H.J., Silva C., Friend R.H., Aida T. (2008). Organogels as scaffolds for excitation energy transfer and light harvesting. Chem. Soc. Rev..

[18] Banerjee S., Das R.K., Maitra U. (2009). Supramolecular gels “in action”. J. Mater. Chem..

[19] Piepenbrock M.-O.M., Lloyd G.O., Clarke N., Steed J.W. (2010). Metal- and Anion-Binding Supramolecular Gels. Chem. Rev..

[20] Dawn A., Shiraki T., Haraguchi S., Tamaru S., Shinkai S. (2011). What Kind of “Soft Materials” Can We Design from Molecular Gels?. Chem.—Asian J..

[21] Dong S., Luo Y., Yan X., Zheng B., Ding X., Yu Y., Ma Z., Zhao Q., Huang F. (2011). A Dual-Responsive Supramolecular Polymer Gel Formed by Crown Ether Based Molecular Recognition. Angew. Chem. Int. Ed..

[22] Tomasini C., Castellucci N. (2013). Peptides and peptidomimetics that behave as low molecular weight gelators. Chem. Soc. Rev..

[23] Tomasini C., Zanna N. (2017). Oxazolidinone-containing pseudopeptides: Supramolecular materials, fibers, crystals, and gels. Biopolymers.

[24] Ishi-i T., Shinkai S. (2005). Dye-Based Organogels: Stimuli-Responsive Soft Materials Based on One-Dimensional Self-Assembling Aromatic Dyes. Supermolecular Dye Chemistry.

[25] Lloyd G.O., Steed J.W. (2009). Anion-tuning of supramolecular gel properties. Nat. Chem..

[26] Yang X., Zhang G., Zhang D. (2012). Stimuli responsive gels based on low molecular weight gelators. J. Mater. Chem..

[27] George M., Weiss R.G. (2001). Chemically Reversible Organogels: Aliphatic Amines as “Latent” Gelators with Carbon Dioxide. J. Am. Chem. Soc..

[28] John G., Zhu G., Li J., Dordick J.S. (2006). Enzymatically Derived Sugar-Containing Self-Assembled Organogels with Nanostructured Morphologies. Angew. Chem. Int. Ed..

[29] Segarra-Maset M.D., Nebot V.J., Miravet J.F., Escuder B., Guggenheim S., van Esch J.H., Gradzielski M., Schalley C.A., Sefcik J., Boekhoven J. (2013). Control of molecular gelation by chemical stimuli. Chem. Soc. Rev..

[30] Lee K.Y., Mooney D.J. (2001). Hydrogels for Tissue Engineering. Chem. Rev..

[31] Shao Y., Jia H., Cao T., Liu D. (2017). Supramolecular Hydrogels Based on DNA Self-Assembly. Acc. Chem. Res..

[32] Xue J., Wang T., Nie J., Yang D. (2013). Preparation and characterization of a photocrosslinkable bioadhesive inspired by marine mussel. J. Photochem. Photobiol. B Biol..

[33] Gong C., Qi T., Wei X., Qu Y., Wu Q., Luo F., Qian Z. (2013). Thermosensitive polymeric hydrogels as drug delivery systems. Curr. Med. Chem..

[34] Shigemitsu H., Fujisaku T., Onogi S., Yoshii T., Ikeda M., Hamachi I. (2016). Preparation of supramolecular hydrogel–enzyme hybrids exhibiting biomolecule-responsive gel degradation. Nat. Protoc..

[35] Alakpa E.V., Jayawarna V., Lampel A., Pé B., Ulijn R.V., Dalby M.J. (2016). Tunable Supramolecular Hydrogels for Selection of Lineage-Guiding Metabolites in Stem Cell Cultures. Chem.

[36] Singh N., Zhang K., Angulo-Pachón C.A., Mendes E., van Esch J.H., Escuder B. (2016). Tandem reactions in self-sorted catalytic molecular hydrogels. Chem. Sci..

[37] Konieczynska M.D., Villa-Camacho J.C., Ghobril C., Perez-Viloria M., Tevis K.M., Blessing W.A., Nazarian A., Rodriguez E.K., Grinstaff M.W. (2016). On-Demand Dissolution of a Dendritic Hydrogel-based Dressing for Second-Degree Burn Wounds through Thiol-Thioester Exchange Reaction. Angew. Chem. Int. Ed..

[38] López C., Ximenis M., Orvay F., Rotger C., Costa A. (2017). Supramolecular Hydrogels Based on Minimalist Amphiphilic Squaramide–Squaramates for Controlled Release of Zwitterionic Biomolecules. Chem.—Eur. J..

[39] Li C., Chen P., Shao Y., Zhou X., Wu Y., Yang Z., Li Z., Weil T., Liu D. (2015). A writable polypeptide-DNA hydrogel with rationally designed multi-modification sites. Small.

[40] Jain M., Matsumura K. (2016). Thixotropic injectable hydrogel using a polyampholyte and nanosilicate prepared directly after cryopreservation. Mater. Sci. Eng. C.

[41] Abbas M., Zou Q., Li S., Yan X. (2017). Self-Assembled Peptide- and Protein-Based Nanomaterials for Antitumor Photodynamic and Photothermal Therapy. Adv. Mater..

[42] Reddy S.M.M., Shanmugam G., Duraipandy N., Kiran M.S., Mandal A.B. (2015). An additional fluorenylmethoxycarbonyl (Fmoc) moiety in di-Fmoc-functionalized L-lysine induces pH-controlled ambidextrous gelation with significant advantages. Soft Matter.

[43] Johnson E.K., Adams D.J., Cameron P.J. (2011). Peptide based low molecular weight gelators. J. Mater. Chem..

[44] Tao K., Levin A., Adler-Abramovich L., Gazit E. (2016). Fmoc-modified amino acids and short peptides: Simple bio-inspired building blocks for the fabrication of functional materials. Chem. Soc. Rev..

[45] Mandal S.K., Brahmachari S., Das P.K. (2014). In situ synthesised silver nanoparticle-infused L-lysine-based injectable hydrogel: Development of a biocompatible, antibacterial, soft nanocomposite. Chempluschem.

[46] Haines-Butterick L., Rajagopal K., Branco M., Salick D., Rughani R., Pilarz M., Lamm M.S., Pochan D.J., Schneider J.P. (2007). Controlling hydrogelation kinetics by peptide design for three-dimensional encapsulation and injectable delivery of cells. Proc. Natl. Acad. Sci. USA.

[47] Li Y., Zhou F., Wen Y., Liu K., Chen L., Mao Y., Yang S., Yi T. (2014). (−)-Menthol based thixotropic hydrogel and its application as a universal antibacterial carrier. Soft Matter.

[48] Van Esch J.H., Feringa B.L. (2000). New Functional Materials Based on Self-Assembling Organogels: From Serendipity towards Design. Angew. Chem. Int. Ed..

[49] Steed J.W. (2010). Anion-tuned supramolecular gels: A natural evolution from urea supramolecular chemistry. Chem. Soc. Rev..

[50] Martin A.D., Wojciechowski J.P., Warren H., in het Panhuis M., Thordarson P. (2016). Effect of heterocyclic capping groups on the self-assembly of a dipeptide hydrogel. Soft Matter.

[51] Misra R., Sharma A., Shiras A., Gopi H.N. (2017). Backbone Engineered γ-Peptide Amphitropic Gels for Immobilization of Semiconductor Quantum Dots and 2D Cell Culture. Langmuir.

[52] Hoshizawa H., Minemura Y., Yoshikawa K., Suzuki M., Hanabusa K. (2013). Thixotropic Hydrogelators Based on a Cyclo (dipeptide) Derivative. Langmuir.

[53] Baral A., Roy S., Ghosh S., Hermida-Merino D., Hamley I.W., Banerjee A. (2016). A Peptide-Based Mechano-sensitive, Proteolytically Stable Hydrogel with Remarkable Antibacterial Properties. Langmuir.

[54] Milli L., Castellucci N., Tomasini C. (2014). Turning Around the L -Phe- D -Oxd Moiety for a Versatile Low-Molecular-Weight Gelator. Eur. J. Org. Chem..

[55] Zanna N., Merlettini A., Tatulli G., Milli L., Focarete M.L., Tomasini C. (2015). Hydrogelation Induced by Fmoc-Protected Peptidomimetics. Langmuir.

[56] Milli L., Zanna N., Merlettini A., Di Giosia M., Calvaresi M., Focarete M.L., Tomasini C. (2016). Pseudopeptide-Based Hydrogels Trapping Methylene Blue and Eosin Y. Chem.—A Eur. J..

[57] Zanna N., Merlettini A., Tomasini C. (2016). Self-healing hydrogels triggered by amino acids. Org. Chem. Front..

[58] Zanna N., Iaculli D., Tomasini C. (2017). The effect of L-DOPA hydroxyl groups on the formation of supramolecular hydrogels. Org. Biomol. Chem..

[59] Zanna N., Focaroli S., Merlettini A., Gentilucci L., Teti G., Falconi M., Tomasini C. (2017). Thixotropic Peptide-Based Physical Hydrogels Applied to Three-Dimensional Cell Culture. ACS Omega.

[60] Ramin M.A., Latxague L., Sindhu K.R., Chassande O., Barthélémy P. (2017). Low molecular weight hydrogels derived from urea based-bolaamphiphiles as new injectable biomaterials. Biomaterials.

[61] Toh W.S., Loh X.J. (2015). Advances in hydrogel delivery systems for tissue regeneration. Mater. Sci. Eng. C.

[62] Zustiak S.P., Wei Y., Leach J.B. (2013). Protein-hydrogel interactions in tissue engineering: Mechanisms and applications. Tissue Eng. Part B Rev..

[63] Liyanage W., Vats K., Rajbhandary A., Benoit D.S.W., Nilsson B.L. (2015). Multicomponent dipeptide hydrogels as extracellular matrix-mimetic scaffolds for cell culture applications. Chem. Commun..

[64] Zhang X., Battig M.R., Chen N., Gaddes E.R., Duncan K.L., Wang Y. (2016). Chimeric Aptamer-Gelatin Hydrogels as an Extracellular Matrix Mimic for Loading Cells and Growth Factors. Biomacromolecules.

[65] Mohanty S., Wu Y., Chakraborty N., Mohanty P., Ghosh G. (2016). Impact of alginate concentration on the viability, cryostorage, and angiogenic activity of encapsulated fibroblasts. Mater. Sci. Eng. C.

[66] Gasperini L., Mano J.F., Reis R.L. (2014). Natural polymers for the microencapsulation of cells. J. R. Soc. Interface.

[67] Focaroli S., Teti G., Salvatore V., Orienti I., Falconi M. (2016). Calcium/Cobalt Alginate Beads as Functional Scaffolds for Cartilage Tissue Engineering. Stem Cells Int..

[68] Du X., Zhou J., Shi J., Xu B. (2015). Supramolecular Hydrogelators and Hydrogels: From Soft Matter to Molecular Biomaterials. Chem. Rev..

[69] Jayawarna V., Ali M., Jowitt T.A., Miller A.F., Saiani A., Gough J.E., Ulijn R.V. (2006). Nanostructured hydrogels for three-dimensional cell culture through self-assembly of fluorenylmethoxycarbonyl-dipeptides. Adv. Mater..

[70] Liang G., Yang Z., Zhang R., Li L., Fan Y., Kuang Y., Gao Y., Wang T., Lu W.W., Xu B. (2009). Supramolecular hydrogel of a D-amino acid dipeptide for controlled drug release in vivo. Langmuir.

[71] Tian Y.F., Devgun J.M., Collier J.H. (2011). Fibrillized peptide microgels for cell encapsulation and 3D cell culture. Soft Matter.

[72] Jung J.P., Nagaraj A.K., Fox E.K., Rudra J.S., Devgun J.M., Collier J.H. (2009). Co-assembling peptides as defined matrices for endothelial cells. Biomaterials.

[73] Peng H.T., Shek P.N. (2010). Novel wound sealants: Biomaterials and applications. Expert Rev. Med. Devices.

[74] Priya M.V., Kumar R.A., Sivashanmugam A., Nair S.V., Jayakumar R. (2015). Injectable Amorphous Chitin-Agarose Composite Hydrogels for Biomedical Applications. J. Funct. Biomater..

[75] Shin H., Jo S., Mikos A.G. (2003). Biomimetic materials for tissue engineering. Biomaterials.

[76] Laurenti M., Al Subaie A., Abdallah M.N., Cortes A.R.G., Ackerman J.L., Vali H., Basu K., Zhang Y.L., Murshed M., Strandman S. (2016). Two-Dimensional Magnesium Phosphate Nanosheets Form Highly Thixotropic Gels That Up-Regulate Bone Formation. Nano Lett..

[77] Kumar V.A., Liu Q., Wickremasinghe N.C., Shi S., Cornwright T.T., Deng Y., Azares A., Moore A.N., Acevedo-Jake A.M., Agudo N.R. (2016). Treatment of hind limb ischemia using angiogenic peptide nanofibers. Biomaterials.

[78] Das A.K., Maity I., Parmar H.S., McDonald T.O., Konda M. (2015). Lipase-Catalyzed Dissipative Self-Assembly of a Thixotropic Peptide Bolaamphiphile Hydrogel for Human Umbilical Cord Stem-Cell Proliferation. Biomacromolecules.

[79] Jacob R.S., Ghosh D., Singh P.K., Basu S.K., Jha N.N., Das S., Sukul P.K., Patil S., Sathaye S., Kumar A. (2015). Self healing hydrogels composed of amyloid nano fibrils for cell culture and stem cell differentiation. Biomaterials.

[80] Liu Y., Ling S., Wang S., Chen X., Shao Z. (2014). Thixotropic silk nanofibril-based hydrogel with extracellular matrix-like structure. Biomater. Sci..

[81] Wu H., Liu S., Xiao L., Dong X., Lu Q., Kaplan D.L. (2016). Injectable and pH-Responsive Silk Nanofiber Hydrogels for Sustained Anticancer Drug Delivery. ACS Appl. Mater. Interfaces.

[82] Ding Z., Han H., Fan Z., Lu H., Sang Y., Yao Y., Cheng Q., Lu Q., Kaplan D.L. (2017). Nanoscale Silk-Hydroxyapatite Hydrogels for Injectable Bone Biomaterials. ACS Appl. Mater. Interfaces.

[83] Mi R., Liu Y., Chen X., Shao Z. (2016). Structure and properties of various hybrids fabricated by silk nanofibrils and nanohydroxyapatite. Nanoscale.

